# Neuroimaging and Brain-Based Markers Identifying Neurobiological Markers Associated With Criminal Behaviour, Personality Disorders, and Mental Health: A Narrative Review

**DOI:** 10.7759/cureus.58814

**Published:** 2024-04-23

**Authors:** Abdulkreem Al-Juhani, Mohammed J Alzahrani, Zainab Abdullah A, Abdulrahman N Alnefaie, Lajeen N Alnowaisser, Wajd Alhadi, Joud K Alghamdi, Moayyad S Bauthman

**Affiliations:** 1 Surgery, King Abdulaziz University Faculty of Medicine, Jeddah, SAU; 2 Medicine, King Abdulaziz University Faculty of Medicine, Jeddah, SAU; 3 Medicine, Alrayan College, Al-Madinah Al-Munawara, SAU; 4 College of Medicine, Taif University, Taif, SAU; 5 Medicine, Almaarefa College of Medicine, Riyadh, SAU; 6 College of Medicine, King Khalid University, Abha, SAU; 7 Medicine, King Abdulaziz University, Jeddah, SAU; 8 Internal Medicine, King Abdulaziz University Faculty of Medicine, Rabigh, SAU

**Keywords:** narrative review, mental health, personality disorders, criminal behaviour, neurobiological markers, brain-based markers, neuroimaging

## Abstract

We begin the review by pointing to the common stigma associated with mental health issues, which often derives from a lack of understanding or incomplete knowledge. Neurobiological research provides us with a new lens to help challenge and dispel common assumptions and misunderstandings and gives an understanding of sexual behaviours that influence society. As such, it generates substantial evidence for the structural and functional asymmetry of the brains of individuals with mental disorders. However, this type of representation poses many challenges to traditional thinking and constantly provokes change in perspective and empathy towards those individuals.

In the review, we go deeper into the effects of neurobiological findings on understanding criminal behaviours and personality disorders, looking further beyond behavioural health. These problems, which were once mainly discussed as moral ones or viewed from the perspective of character flaws, are analysed today through neurological considerations pointing to their complexity. When the root of bipolar disorder is revealed to be neurological, society will react with more information and understanding, hence reducing the stigmatisation and discrimination meted out to people with these problems.

At a macro level, findings from neurobiology affect society in ways that go beyond individuals; social attitudes, laws, and policies about the services rendered are influenced. Operating as a catalyst within the community, neurobiological research helps to initiate social change through the creation of an informed, understanding public forum. Thus, it creates broader value for those dealing with behavioural and mental health challenges.

The first and most important question of this narrative review is focused on identifying identifiable neurobiological markers that are closely related to criminal conduct, personality disorders, and mental health disorders. Through this review, we aim to present detailed insights into the neurological foundations that anchor these phenomena via a narrative analysis of contemporary literature. The potential implications are finding problems early to apply specific treatment and learning an advanced strategy for social attitudes. This will promote a more humanistic approach based on adequate information on the behavioural and mental health issues involved.

## Introduction and background

Uncovering the neurological basis of criminal behaviour disorders, personality disorders, and mental health is essential within modern neuroscience and psychology [1]. The complexity of these phenomena has attracted researchers and experts in the study of human behaviour and mental states. This introduction emphasises the significance of understanding the intertwined relationship between neurobiology and behaviour, highlighting its implications for preventive strategies, diagnostic procedures, and intervention measures [2].

The emergence of novel neuroimaging techniques such as functional magnetic resonance imaging (fMRI), positron emission tomography (PET), electroencephalography (EEG), and neurobiological markers has brought about a remarkable shift in perspective. Scientists have now advanced methods to explore the brain regions associated with behaviour and mental health, offering the potential for a deeper biological understanding of these complex phenomena [3]. Understanding the neural basis of behaviours allows for the development of screening tools to identify at-risk individuals before obvious symptoms appear [4]. Furthermore, the act of identifying facilitates the development of specialised treatment methods that target the root cause rather than merely relieving symptoms [5].

This shift allows for a more in-depth investigation of the molecular mechanisms behind these events as well as the potential to inform better prevention, diagnosis, and treatment strategies [6]. The integration of neuroscience and psychology through advanced neuroimaging technologies offers new perspectives on human behaviour and mental health, presenting opportunities for systematic and targeted approaches to complex problems in criminality, personality disorders, and global mental health issues and reducing the stigmatisation and discrimination meted out to people having these problems.

## Review

Significance of neurobiological markers

Neurobiological markers refer to measures that can be detected in either the structure or the operation of the neurological system. They represent an important way of understanding and addressing some behaviours, diseases, or mental states [7]. In many areas, such as early detection and intervention, these markers’ discovery has major implications.

Prompt Detection and Intervention

Creating neurobiological markers related to criminality, personality disorders, and mental health problems may initiate a new era of early diagnosis and treatment. The fact that the neurological process and behaviour often correlate means that detectable indicators can happen before any obvious symptoms to help identify people at risk before disease onset [8]. Early diagnosis allows for the implementation of individual intervention methods that may change the course of these disorders and can lead to an improvement in people’s state and society as a whole [9].

Interventions that were implemented early from the recognition of neural indicators could be presented in a variety of ways [10]. For instance, a person with neurobiological markers of those at risk may be offered targeted cognitive or behavioural therapy that aims to reorganise specific neural circuits associated with risky behaviours [7]. Besides, pharmaceutical treatments can be tailored depending on neuromolecular imbalances or abnormalities identified through neuroimaging or biomarker studies [8]. The introduction of neural knowledge into present early intervention schemes can have the potential to strengthen the effectiveness of preventive strategies, thereby transforming the field of mental health and behavioural therapies.

Focused Therapeutic Strategies

The neural perspective enables scientists to identify the exact mechanism underlying a behavioural disorder. This could revolutionise the field of targeted treatment and aid in identifying brain dysfunctions related to impulse control and decision-making in criminal behaviour [3,8,11,12].

From the neural analysis, it is crucial to understand how treatment strategies guide personality disorders. The development of clear brain circuits that correlate with maladaptive personality traits also leads to a greater understanding of the neurobiology of these disorders [5]. Using this knowledge, treatment approaches can be designed, targeting the mentioned brain circuits to provide a more personalised and effective treatment method [8]. Neuromolecular indicators can be utilised by clinicians to develop personalised treatment regimens that would target the unique brain profiles of personality disorder-affected persons.

Such a transition from general therapies to focused ones merges harmoniously with the overall trend of medicine towards personalised and precise approaches [9]. Personalising interventions based on neuromolecular platelets enables therapies to be brought in line with the individual’s brain functioning. The individualised approach not only improves the effectiveness of the therapies but also minimises the likelihood of encountering side effects because treatment precisely addresses the specific neurobiological characteristics of each individual [10]. Moving from the uniform to the non-stationary model has considerable potential for significant improvement in treatment outcomes and, ultimately, the standard of care in the mental health arena [12].

The use of neurobiologically inspired therapies for the treatment of behavioural and mental health disorders marks a watershed moment in the history of our attempts to address this wide array of illnesses. The conventional models often emphasised symptom management but did not meticulously examine the brain workings involved [13]. The neural insights help provide a deeper understanding to enable the creation of therapies that focus on the root factors contributing towards behavioural and mental health complications [6].

Researchers have made significant progress in understanding drug use disorders by studying neural indicators associated with reward pathways and neurotransmitter systems. This understanding has provided an opportunity to establish pharmacotherapies that target the neural markers linked with drug addiction [7]. With these therapies, practitioners try to overcome cravings, restore balances in neurotransmitters, and help patients win over addiction [8].

As far as the designing of therapies for mood disorders is concerned, neuromolecular indicators play a major role in this regard, especially when it comes to cases of depression and anxiety [11].

The understanding of this information has shaped the formulation of stimulant drugs targeted at specifically correcting imbalances in neurotransmitters of the prefrontal cortex (PFC), which improve attention and control over impulsive behaviour [14].

The trend towards specific therapies, however, holds promise for the field of neurodegenerative diseases, including Alzheimer’s disease, that pose major challenges [15]. The information may appear crucial for accurate diagnoses because some neurobiological markers connected to the accumulation of beta-amyloid plaques and neurofibrillary tangles in individuals’ brains are used [16]. The identification of such markers has enabled scientists to develop disease-modifying drugs, offering intervention points that aim to slow or stop the progression of neurodegenerative mechanisms [6].

This psychotherapy, which uses principles from neurobiology, allows an intervention with increased levels of precision and efficacy as a result of the general aim of psycho-specific medicine [8].

As shown earlier, there is potential to use the same neurobiological therapies in preventive initiatives, which will make them similarly effective. Identification of at-risk persons or early neuromolecular indicators can open the door to early preventive therapies to avert the onset of behavioural or mental health disorders [11]. The move towards prevention is consistent with a preventive approach to mental health treatment that requires early intervention and targeted interventions designed to reduce risk factors [12].

Despite the potential benefits of neurobiologically based therapies, their practical implementation presents several challenges [17]. Nonetheless, careful and cautious treatment is necessary, given ethical considerations over the use of neuromolecular data, fears over stigmatisation, and the need for judicious application in legal settings. At the same time, there is a risk that adopting an excessively neuromolecular perspective may result in an oversimplification of the complex interplay among genetic, environmental, and psychological variables that contribute to behavioural and mental health problems [14].

Neural knowledge applied to the development of particular treatment approaches is one of the major changes in focus within this area of psychiatry. This approach helps in acquiring a more refined understanding of the brain mechanisms that underlie specific behaviours and pathologies, which provides therapies that target the underlying causes rather than merely alleviating symptoms [15]. The development of personalised and precision medicine goes together with the overall tendencies in medical practice, opening new doors for more efficient and personal means of treatment that can work in various cases of behavioural and mental health problems [16]. Neurobiology advances can improve mental health care significantly, which will be accomplished through better treatment results, better patient experiences, or a deeper understanding of the complicated brain-behaviour link.

Mitigating Stigma and Correcting Misconceptions

Neuromolecular study has the potential to lead to drastic changes and further social stigmas as well as misconceptions about criminal behaviour, personality disorders, and mental health [18]. However, neural discoveries can bring benefits by extending our awareness of these phenomena with a more detailed and compassionate approach that might help reduce the stigma and build better social support networks [19].

The social stigma associated with mental health disorders has been identified as one of the long-standing challenges in addressing the issue. Mistrust and false facts regularly lead to social phobia, so those who need help face barriers [20]. Neuromolecular studies must be at a high level to shatter these myths by providing empirical evidence about the biological bases of mental health disorders [21]. Neuroimaging techniques, such as diffusion tensor imaging (DTI) and tractography, underline the differences in brain structure and functioning in mentally ill people and are valid instruments for challenging current prejudices [22].

Neurobiological perspectives infiltrate into different aspects of human life, not only mental health disorders but also criminal behaviour and personality disorders [15]. Moral judgements and punishment are often associated with societal response, among other aspects, to criminal behaviour and personality disorders [23]. However, understanding the neuroscientific basis of these challenges allows for a more informed and considerate social response.

By changing our viewpoint from a partial morality or character view to recognising the neuromolecular factors involved, we may gain a broader view of criminal behaviour and personality disorders. This acceptance underlines the sophisticated nature of these issues [16]. This refined understanding can lead to the creation of structures that are more humane and supportive, rendering persons who are struggling with these problems less marginalised and stereotyped [18].

Basically, emphasis on neural findings to combat stigma not only affects individuals but also affects social norms, public policies, and support systems [19]. Neural research is also significant because it offers more than mere revelations for therapeutic treatment to be implemented. It also has the potential to shape debates and challenge deep-rooted social assumptions [20].

The purpose of this investigation is to reveal the complex relationship between the neurological system and human conduct, which does more than reveal symptoms apparent in criminal behaviour, personality disorders, and mental health issues [23]. By analysing the existing literature, with this review, we intend to present general insights into the neural substrates that give rise to these phenomena. The results of this review can thus shed light on the phenomena in question, with some important implications for early identification, interventions, and societal approaches [24].

The cogency of neurobiology with criminal activity is especially applicable in interrogating cultural biases [25]. Criminal behaviour can be interpreted from its conventional perspective, where morality is highly emphasised and individual responsibility is stressed, rather than the possible underlying biological influences. The neuromolecular approach is contrary to such a stance because it focuses on the part that brain systems play in behaviour [26].

The neural conceptualisations not only affect change in the perception of personality disorders but also contribute to changing cultural outlooks. First, these diseases have been prone to stigmatisation and misunderstanding, through which people have been judged characteristically [27]. In contrast, the interpretation of implicated brain structures can provide another angle and prove that personality disorders are not only due to individual failures but also have biological backgrounds. Through the change of perspective, social disapproval can be reduced, allowing people to adopt a more compassionate attitude that is understanding in nature and enabling greater empathy for persons suffering from personality disorders [28].

Neural concreteness has been proven to produce changes in brain structure and function, confirming the biological nature of various diseases. Such comprehension triggers a sense of understanding and support, which is essential for compassion rather than condemnation [29].

The implications of this narrative review, however, are not necessarily limited to addressing stigma but rather act as effective tools in aiding early detection while also identifying specific therapies. The early identification of these cues could have a tremendous impact on prevention and intervention strategies by enabling immediate and accurate treatment of people at risk [9].

Neuromolecular research is becoming stronger in eliminating cultural stigma concerning criminal behaviour, personality disorders, or mental illness. Neural insights add depth to the understanding and allow people to be more sympathetic in their apprehension by providing empirical evidence for the biological base of these phenomena. The outcomes, however, are not confined simply to research but also include social values, laws, and support systems. As we try to unravel the mystery of the role of neurobiology in our behaviour, it seems quite clear that a deep understanding of this phenomenon promises to bring significant transformations to society [12]. Such an understanding contributes to a greater sense of empathy and acceptance of people with behavioural and mental health concerns, thus creating hope for positive transformation to explore neural markers associated with personality disorders, mental health, and criminality. This comprehensive meta-review is expected to expand on the current knowledge at the crossroads between neuroscience and behavioural sciences, thus paving the way for a deeper understanding of the molecular mechanisms underlying human behaviour and mental health.

Summary

Neuroimaging Techniques

According to Klaus et al., neuroimaging techniques play a significant role in understanding the anatomy and physiology of the human brain, but further studies are needed [1]. Despite shortcomings such as poor temporal resolution, MRI can provide a detailed visualisation of different brain structures [4].

PET is an effective imaging method for neuro examinations. The process involves injecting a positron-emitting radiopharmaceutical into the circulatory system, whereby it releases positrons that combine with neighbouring electrons to generate gamma rays [3]. The process of PET scans is to record and analyse the gamma rays to get information about particular localisation of blood circulation and metabolic activity in the brain [4]. The ability of PET to evaluate brain functions gives it superiority, making it useful for the study of diseases such as Alzheimer’s disease. However, it is also worth mentioning that this approach has several limitations, such as the lower spatial resolution characteristic compared to the usage of MRI and the possible risk of irradiation [5].

fMRI is the modern method used in the study of brain activity, with the principal mechanism referring to sensing changes in blood flow and oxygenation levels [3]. The technology is highly precise in its spatial resolution, which serves to link brain areas with the respective functions. However, the fMRI technique has limitations, which include its reliance on haemodynamic responses to approximate indirect brain activities, thus inducing a lag in recording real-time neuronal events [4].

According to Carbia et al. [3], DTI is an important tool for understanding the anatomy and connectivity of white matter pathways in the brain. The DTI is a diffusion MRI technique used to study the anatomical connections in the brain; that is, it gives information on how water molecules pass along the nerve fibres [4]. The main benefit of this approach is the possibility to precisely follow the roads of white matter in the brain, which facilitates research on various neurological disorders including schizophrenia. Limitations may include problems in the correct interpretation of overlapping fibres and also susceptibility to artefacts [5]. EEG, the recording of electrical activity from the scalp, presents an accuracy surpassing all other methods for determining time. The technique of EEG is fundamental when investigating the activities of the brain, especially in studying cognition and determining neurological conditions such as epilepsy. Noordermeer et al. [8] described the shortcomings of this method as poor spatial resolution and susceptibility to artefacts caused by factors that do not originate from the source.

Storvestre et al. [9] described magnetoencephalography (MEG) as a technique that enables the measurement of magnetic fields emitted by neurons. Dynamic processes in the brain can be recorded in real-time using this method. MEG has several advantages, including high efficacy in spatialising fast neuronal activities with accuracy at the timing level, as described by Storvestre et al. [9]. However, low spatial resolution and susceptibility to external noise are the major limitations of this technology [4].

It is noteworthy that each neuroimaging approach has its own strengths and weaknesses with regards to the study of the brain. Typically, scientists use one of these approaches or a combination to gain better insight into brain anatomy and function [3,4]. The choice of methodology depends upon the particular questions from the study and at the same time on trade-offs among geographical and temporal accuracy, intrusiveness, potential risk, and so forth.

Neurobiological Markers in Criminal Behaviour

Cremers et al. established that the efforts towards studying biological markers associated with criminal behaviour have been a complex undertaking [5]. This is because researchers have used various neuroimaging approaches to explore the interactions between brain activity and propensity for criminality [8]. In all of these studies, it is clear that changes are occurring in some specific parts of the brain. The cases of abnormalities in the PFC, which is responsible for decision-making and inhibiting impulses, can serve as a good example. These deviations consistently appear in connection to some concerns [9]. Researchers using MRI techniques have identified certain anatomical abnormalities such as thinness of the PFC volume, specifically in individuals displaying criminality. This finding corresponds to the hypothesis that the executive processes have some relation to deficiencies of processing in the PFC and impulsive and antisocial behaviour [3].

Cupaioli et al. conducted experimental research based on PET and fMRI to understand the functional aspects of the brain [4]. The information that has been gathered from this investigation is beneficial in the discovery of the neurological processes involved with criminal behaviour. The amygdala in the limbic system has been noted as a common pattern, and anomalies in the amygdala have also been described [3]. The dysfunction in the amygdala has been associated with hyperactivation or altered connectivity, which reduces emotional reactivity and diminishes empathetic responses, respectively, leading to violent and antisocial behaviours [4]. In addition, differences in the striatum, a region of the brain that is linked with the giving out of rewards, are also observed. The aforementioned findings suggest a positive relationship between impaired reward systems and underlying criminal tendencies [5].

In the study of criminal behaviour, fundamental studies have illuminated the connection between genes and neurobiological markers, drawing attention to genetic determinants [8]. Researchers have revealed correlations between monoamine oxidase A (MAOA) variants, which contribute to regulating neurotransmitters including serotonin, and impulsive or violent behaviour [9]. The combination of genetic and neuroimaging information has provided a more complex understanding of how different genetic variations may possibly affect brain connectivity, which would be reflected in an increased criminal inclination [3].

Storvestre et al. reported that one of the results of the use of DTI was the detection of disturbances in connectivity between different brain areas in individuals with criminal backgrounds, which signalled lowered white matter integrity [9]. The disruption of structural integrity has been associated with impairment in the communication between widespread parts of the brain, which leads to emotional regulation and impulse control. Cupaioli et al. have posited that structural deviations can cause cognitive and emotional impediments associated with criminal behaviour [4].

The application of EEG and MEG in studies has contributed meaningful knowledge regarding time cycles for people who are predisposed to criminal behaviour [9]. As for the population showing criminal behaviour, abnormal patterns of brain oscillations were revealed in regions associated with inhibitory control [9]. The findings show disruptions in the networks of impulsive responses, which again support the claim that criminal behaviour involves neuromolecular factors [10].

The findings from these studies reveal disparate neurobiological markers associated with criminal behaviour, underlining the need to appreciate the diverse nature of this population [12]. The neural discoveries presented in the intricate array are influenced by the variation in degrees and types of criminal behaviour, comorbidities, and features of the context [13]. In addition, ethical issues, the difference in the number of samples, and the research methods used are hindrances to forming a conclusive narrative [6]. The increasing quantity of neuroimaging data presents a focus on the capability to understand criminal behaviour from a neurobiological point of view. This supports the establishment of targeted treatments and rehabilitation programmes that are grounded in a deeper understanding of the involved brain processes [7].

Neurobiological Indicators in Personality Disorders

Noordermeer et al. demonstrated that research on neurobiological markers associated with personality disorders has led to valuable outcomes regarding the neural basis of such complicated and diverse conditions [8]. Studies involving multiple neuroimaging techniques have repeatedly demonstrated specific alterations both in the form and function of the brain, which are associated with personality disorders [11]. A characteristic feature of individuals diagnosed with antisocial personality disorder (ASPD) is the presence of anatomical anomalies, especially in the ventromedial prefrontal cortex (VMPC). The prefrontal region is an important area serving moral judgement, inhibition of impulses, and emotion regulation. This implies that there is an association between the defective prefrontal circuitry and the impulsive and antisocial behaviours identified with ASPD [12].

Neuroscience research on borderline personality disorder (BPD) has resulted in the discovery of abnormalities in cortical regions, specifically the limbic system areas such as the amygdala and hippocampus [17]. The characteristic research result of fMRI in BPD is the increase in activity of the amygdala and dysfunctional network connections [14]. These results imply that individuals with BPD have a certain level of being unable to control their emotions and also are highly vulnerable to stressors. Despite its involvement in memory and emotional processing, the hippocampus shows structural abnormalities in BPD [15]. The latter adds to the hyperexcitability of emotional reactions as reported in patients with BPD [16].

It has been shown by DTI that patients with different personality disorders are in a disconnected state using the analysis of white matter integrity by Xu et al. [18]. To date, consistent evidence has demonstrated that BPD is associated with integrity pathology of the cingulum bundle, a white matter tract interlinking the frontal and limbic regions [4]. People who have been diagnosed with narcissistic personality disorder (NPD) tend to have structural changes in the white matter tracts involved in self-reference and control of emotions [3]. The presence of these white matter anomalies could also be a factor contributing to the challenges that individuals face in their interpersonal relations and have implications for emotional regulation [4].

Genetic factors are associated with the development of neurobiological markers that may cause personality disorders [5]. In the case of investigating the genetic origins of BPD, Noordermeer et al. indicated that abnormalities were found in genes associated with the serotonin and dopamine pathways, affecting brain mechanisms in mood regulation and impulsivity [8]. The mechanism by which genetic predispositions interact with neuromolecular signs overlaps with the complexity of the understanding of personality disorders, highlighting how a multimodal approach is required [9].

Psychopathology investigations using EEG techniques have thus shown the characteristic brain wave patterns of personality disorders. Individuals diagnosed with avoidant personality disorder (AvPD) show a higher level of frontal alpha asymmetry in comparison with other people, reflecting altered tendencies towards approaching and retreating from the situation [9]. Compared with the findings in normal subjects, EEG results in obsessive-compulsive personality disorder (OCPD) patients show high frontal theta activity that corresponds to improved cognitive control and increased rigidity associated with thought patterns [10].

The neurobiological indicators in people with schizotypal personality disorder (SPD) were studied, and it was found that there is variation in brain areas associated with social cognition and perception [12]. Structural alterations in the superior temporal gyrus and fusiform gyrus are considered up-to-date findings, which are responsible for processing social information or facial expressions [13].

The studies cited here highlight common traits found in brain biological markers associated with personality disorders. Yet, it is worth noting the variety of individuals within each diagnostic group [6]. The neuromolecular landscape is defined by the high degree of symptom intensity variability, the level of comorbidities, and individual differences. In addition, the various study methods, limited samples, and the fact that replication is problematic demonstrate the need for continued studies so that our understanding of brain connections with personality disorders is improved [7]. Integration of neurobiological findings with environmental factors and psychosocial aspects has the potential to contribute to a broader and deeper understanding of the aetiology and phenotypic expression of personality disorders [8].

Neurobiological Indicators in Mental Health

Exploring the neurobiological markers associated with mental health disorders has been a crucial avenue of research that helps us understand the biology behind different illnesses. Several studies have shown certain consistent neuromolecular changes in psychopathology in many mental health disorders [11]. There is a wide range of detailed investigations on depression as one of the most widespread mental diseases that cause a striking change in the structure of the hippocampus and amygdala. Schiffer et al. and da Cunha-Bang et al. noted that these brain areas are of great significance because they determine the level of emotion regulation [12,17]. In addition, research using fMRI has shown consistently increased default mode network (DMN) connectivity when an individual is at rest, which implies disorders in the processing of self-referential information. Likewise, findings that have been reported to be common in anxiety disorders have also reflected similar reports, pointing out a common brain circuitry that participates in emotion processing and regulation [14].

Neural research has been intensive in schizophrenia, a mental disorder whereby discernment and sensory perception are distorted [13]. Although the structural abnormalities of the PFC, thalamus, and hippocampus are revealed by MRI investigations, they are frequently associated with bipolar disorders [15]. The schizophrenia-related PET studies emphasise the role of neurotransmitter imbalance in this disorder, and functional anomaly in the dopaminergic system is demonstrated through PET studies of schizophrenic patients. In addition, studies have shown DTI disruptions in white matter pathways, signifying poor communication in core regions of the brain associated with the illness.

Bipolar disorder, which has been defined by alternating phases of depression and mania in several studies [6], always exhibits neurobiological changes. Structural MRI studies show that both the amygdala and hippocampus, as well as the PFC, are altered when compared to controls, thus pointing out the role of limbic and prefrontal areas in mood control [7]. Researchers using functional imaging techniques such as fMRI and PET have observed repeated changes in specific brain areas in mood episodes. Genetics is also important as researchers have demonstrated the existence of genetic differences associated with neurotransmitter paths and circadian rhythm control.

PTSD, one of the conditions that often result from traumatic events, is known to be associated with particular neuromolecular signs [8]. Neurobiological researchers, with the help of neuroimaging tests, fMRI, and PET, show the changes in the amygdala and prefrontal cortex, which leads to disturbances in fear processing and emotion control. FDG-PET scan shows dynamic changes in the metabolic activity of the hippocampus, and evidence from structural MRI reveals consistent oddities in hippocampus volume, implying a link between traumatic stress and brain areas adjacent to memory [11].

In a neurobiological investigation of obsessive-compulsive disorder (OCD), Schiffer et al. identified several brain circuits associated with the condition [12]. In this narrative review, functional MRI studies consistently centre on demonstrating the role of the cortico-striato-thalamo-cortical (CSTC) circuit in obsessive-compulsive symptoms development. This emphasises the contribution of disturbed communication between PFC and basal ganglia in the occurrence of these signs [17]. The fact of structural deviations in the orbitofrontal cortex and the caudate nucleus, revealed by the MRI method, further proves the part these brain structures play in the development of OCD [14].

Attention-deficit hyperactivity disorder (ADHD) is described as a disorder involving difficulties and characteristics of attention, hyperactivity, and impulsiveness [15]. The neuroimaging researchers note variations across the structure of the PFC, basal ganglia, and cerebellum. Functional imaging studies, including those involving fMRI, show disturbances in the workings of the DMN and frontoparietal networks that contribute to cognitive impairments due to ADHD, giving neurological manifestations of ADHD [16].

One consistent finding in the results indicates the interrelation of neurobiological alterations in different mental health illnesses [18]. Consistent disorders in the limbic areas such as the amygdala and hippocampus demonstrate the importance of emotion processing and regulation to well-being. The occurrence of abnormalities in the PFC neuroanatomical structure, which is connected to executive processes and cognitive control, is reported frequently. In addition, the involvement of different brain pathways, that is, the CSTC circuit, suggests that various disorders have common neurobiological bases [18].

Despite these parallel reports, it should be recognised that each disorder in mental health is completely different in that individual differences, comorbidities, and the environment have magnified the manifestations of such diseases [19]. The differences in methodology, which can include the number of subjects, different imaging techniques, and qualifying criteria for diagnosis, can sometimes lead to difficulties in matters such as integration into a full understanding of neurobiological markers. Marrying neural advances with genetics, environmental factors, and psychosocial variables can yield a wider understanding of mental health disorders and design therapies based on neuromolecular principles.

Synthesis

Combining research findings from three domains, such as criminal behaviour, personality disorders, and mental health, reveals close links between neurobiological markers and the form of these complex manifestations [20].

When a person displays criminal behaviours, then the neuromolecular indicators associated with impulsive and antisocial conduct are quite consistent. It has been observed that people with criminal leanings have structural defects of the PFC, especially the VMPC. This type of malfunction, which is critical for decision-making, control, and restraint of impulses might be involved in criminal acts characterised as impulsive and violent. In addition, findings on neuroanatomical abnormalities in the limbic system, specifically the amygdala, highlight the role of emotion dysregulation in crime. Findings from the research show that there are disturbances in the reward processing system, specifically in the striatum, which factor in flawed reward processes, leading to criminal proclivities [21].

The PFC, which is responsible for impulse-control regulation and ethical decision-making, consistently demonstrates dysfunction in individuals diagnosed with ASPD and NPD [23]. Changes in the white matter result in an impairment of connections between some important brain areas, consequently affecting emotional regulations and interpersonal interactions. Genetic factors, specifically the differences in serotonin and dopamine pathways, interact with neurobiological markers, causing the development of personality disorders [24].

If we consolidate investigations of mental sicknesses, it reveals common neuromolecular changes in numerous illnesses. There are key structural changes in the hippocampus and amygdala in depression and PTSD [25]. fMRI identifies a disruption of the DMN as a potential dysregulation connectivity that is equally shared by both depression and anxiety disorders, which could be a major anomaly in self-referential processing. The CSTC network is implicated in OCD and ADHD, thus pointing to the disturbance of different brain networks that contribute to both diseases [26].

Combining information from different domains reveals broad trends in neurobiological markers. The limbic structures play a role in many of the symptoms identified as being found in criminal behaviour, personality disorders, and mental health conditions. Such symptoms include emotional dysfunctionality, fear processing alterations, and performance-based memory problems [30]. In addition, we even find persistent observations of anomalies in the PFC, which is responsible for executive operations, decision-making, and impulse control.

The fact that there are definite individual pathways, such as the CSTC circuit and the DMN, reflects common neurobiological substrates found in many situations [27].

Genetic factors play a role in shaping the development of neurobiological markers across three domains, criminal behaviour, personality disorders, and mental health, because heterogeneity is observed within each domain due to such influence. Genetic issues arising from variations in the pathways of neurotransmitters, especially those associated with serotonin, dopamine as well as MAOA, react with environmental factors to influence the development and incidence of criminal acts, personality disorders, and mental health issues [31].

Despite these commonalities, it is necessary to identify the other features present within each domain. Because of the great number of socio-environmental factors, as well as individual choices, which medically determine criminal activity, neurobiological markers of these criminal acts may also vary greatly, reflecting the wide spectrum of activities categorised as illegal [18]. Personality disorders demonstrate specific alterations in the brain structure that are consistent with certain maladaptive features constituting persistent patterns of behaviour and signs. Mental health problems are a broad range of ailments that present unique signs and origins of the disease. They include a multidimensional neuromolecular markers set [17].

Finally, the synthesis of neurological studies connected to criminal actions, personality disorders, and mental health conditions points out some similarities and differences. Some of the prevailing issues are related to malfunctioning within the limbic areas, anatomic lesions in the PFC, specific brain circuits, and heritable factors. However, the more we learn about these neurobiological markers, the better we understand where and how intricate psychological processes originate and appear. With the integration of neural findings with other factors such as genetics, environmental influences, and psychosocial concepts, there is a possibility of personalised interventions and focused therapies based on a detailed understanding of the brain processes involved [10].

The significance of neurobiological markers revealed in connection to criminology, personality disorders, and mental diseases such as depression cannot be underestimated because they help us to understand and solve them properly. An in-depth analysis of the literature by researchers in these fields shows how many opportunities are there for targeted treatments, preventive measures, and more advanced care for mental health issues [3].

Starting with the illegal behaviour, neuromolecular markers are identified, which provide a foundation for understanding the underlying mechanisms that contribute to impulsive and antisocial behaviour. The analysis of the neural structural variations in the PFC and the limbic system can give insight into cognitive, emotional, and behavioural dysfunctions associated with criminality (Roelofs et al., 2019). Having this comprehension in mind can help guide proactive initiatives and treatments focused on improving impulse control and decision-making skills. The neurobiological orientation can enhance the rehabilitation programmes through the inclusion of methods based on it. These interventions should be geared towards cognitive-behavioural therapies aimed at specific deficiencies in executive function, which are related to criminality. In addition, the legal systems may also be able to use neurological data in the process of assessing threat and sentencing, therefore fostering a more informed and sophisticated approach to the administration of criminal justice (Pfeifer et al., 2005).

In personality disorders, neuromolecular indicators demonstrate a variety of potential impacts, which provide information on possible treatment goals and intervention strategies. Structural changes in the amygdala and hippocampus found in BPD patients show an apparent need to develop therapeutic approaches to regulate emotional reactions and enhance emotional robustness. Such cognitive-behavioural treatments aimed at emotion regulation and dialectical behaviour therapy, which is explicitly aimed at interpersonal issues, actually have substantial compatibility with the adopted neurobiological markers [26]. If the executive dysfunction is caused by anomalies in the PFC, targeted interventions for the condition may have proven benefits for diseases such as ASPD. Adding neural data to treatment modalities may make it possible to increase the efficacy of the treatments and tailor sessions based on specific brain dysregulations [30].

Genetic correlates of personality disorders signify the importance of personalised treatment methods [27]. Therefore, the discovery of specific genetic variants related to serotonin and dopamine pathways opens paths for pharmacological approaches aimed at these particular neurotransmitter systems. The use of psychopharmacological therapies in combination with psychotherapeutic interventions can result in a comprehensive treatment plan to control the symptoms, while also addressing the neural aspects underlying personality disorders [28].

Understanding the neurological markers is of great importance for developing the ability to diagnose mental health problems, design specific treatments, and increase precision medicine methods, which are popular in this field [32]. If the depressed person has changes in the hippocampus and amygdala, then therapeutic options may be suggested. Other therapies that can be conjunctive treatments for those with transient depressive states include neuro-stimulation techniques such as transcranial magnetic stimulation or electroconvulsive therapy [33]. These approaches are aimed at changing the patterns of brain activity. In addition, finding alterations in neurological disorders of anxiety within the DMN could help in producing therapies that target these networks directly to increase the potency of a treatment [29].

Effective interventions that target the flaws in the PFC and the dysregulation of dopamine may prove beneficial to an individual with a case of schizophrenia characterised by hallucinations and cognitive disruptions [34]. The cognitive remediation treatments, which target better performance of cognitive processes and higher executive functions, are concordant with the neurobiological markers discovered in schizophrenia. These pharmacological therapies, based on neural research, are a key aspect of schizophrenia treatment and include antipsychotic medications aimed at dopamine receptors [31].

Identifying the brain networks that contribute to OCD and ADHD leads to new avenues for specific treatment options. Other neuromodulation therapies that specifically target the CSTC circuit emerge as alternative therapy approaches for OCD. Changes in the PFC related to ADHD can be seen as effects of interventions aimed at bettering executive functioning and attentional control [31]. Non-pharmacological therapies, including cognitive training and behavioural interventions, can also be used with standard medicines to offer a multipronged approach to tackling such diseases [35].

The availability of genetic variants associated with mental health disorders facilitates the possibility of utilising precision medicine strategies. Pharmacogenomic testing is a practice of analysing an individual’s genetic makeup to predict the response of that person when exposed to drugs [29]. These data can be used to make well-informed decisions concerning the drugs being prescribed and also to correctly determine the appropriate dosage. This approach allows us to consider a person’s genetic inclinations and prevent undesirable effects, increasing the possibility of achieving positive results [3].

Regardless of all these positive implications, it is crucial to avoid the abuse of neurobiological information. However, the ethical ramifications of the application of neural data in the court system, the threat of social tagging, and the possibility of overgeneralisation of complex situations require careful treatment [36]. In addition, each diagnostic category includes heterogeneity of characteristics, which calls for an individualised and holistic approach [37].

There are far-reaching implications associated with the availability of neurobiological markers of criminal behaviour, personality disorders, and mental disorders. These entail the importance of identifying specific treatments, adopting a preventive stance, and pursuing a more sophisticated approach to mental health care [30]. The neural knowledge can be integrated into treatment protocols, which can help enhance the therapy’s accuracy and efficacy by enabling a point where these problems are being handled in more customised patient-centred ways [27]. With further scientific inquiry into the realm of neurobiology, an increasing opportunity for innovative and personalised approaches to understanding and managing mental ill health arises. This provides the solution to improving outcomes and promoting radical well-being [38].

By their nature, methodological constraints are inherent in the studies carried out on criminal behaviour, personality disorders, and mental health illnesses. Thus, it is essential to analyse these limitations critically to ensure that the findings are satisfactorily sound and credible. A narrative literature review enables us to identify common issues to be addressed and provides invaluable information on potential directions that future researchers can take to address these obstacles holistically [39].

Criminal behaviour is the phrase that refers to various criminal acts from minor offences such as shoplifting to the most severe crimes such as homicide and rape. The diversity is a problem when one tries to identify specific neurobiological markers [28]. Future researchers should strive to classify crime based on its sub-categories such that comprehensive neurobiology analyses of criminal acts can always be easily conducted. In addition, population differences across studies may arise from different legal definitions and cultural phenomena that constitute crime in various countries. It is possible that future studies could be enhanced if there is some form of cooperation between nations to ensure cross-cultural validity and generalisability of findings [32].

The variability of personality disorders may be a sign of the limited scope of the current research and the challenges in determining specific neurobiological indicators for each condition [40]. Reliance on symptom severity and the presence of comorbidities for the heterogeneous sample of individuals with various personality disorders makes it challenging to determine unique markers associated with each type [33]. For the sake of the quality improvement of future studies, it is advisable to use uniform samples. Sometimes, these can be attained through longitudinal designs that track individuals from the early stages of the condition to its full manifestation [35]. In addition, if we compare how the symptoms are shared by the different personality disorders, we can develop a better understanding of the specific neurobiological features that characterise each illness. It is possible to use dimensional approaches that include all personality and character disorders because doing so may increase the accuracy of neural studies [29].

Genetic factors play an important part in understanding neuromolecular markers, but as far as the method is concerned, they have some issues regarding the representative nature of the samples and the complicated issue of gene-environment interactions. This generalisability of research findings in many genetic studies is significantly limited because their authors use small sample sizes [34]. In a bid to enhance the validity of genetic correlations, future researchers should concentrate more on large-scale collaborative efforts that serve to boost statistical power. To have a clearer picture of how genetics and the environment interact in shaping neurobiology, it is crucial to incorporate detailed assessments of environmental factors, such as early life experiences and trauma, in the assessment of gene-environment interactions [31].

The two broad methodological challenges in mental health research are the diversity of diagnostic classifications and the need for self-reported assessments. The appearance of the same symptoms in various mental health problems could present a problem of accurate identification of different neural symptoms [41]. The next step in the research is to study trans-diagnostic approaches, which are aimed at defining the common material of various diseases and elucidating shared underlying neurobiological mechanisms [42]. Furthermore, the representation of objective signals, such as biomarkers or physiological indices, may increase the validity and accuracy of outcomes with no requirement for self-reported data.

Despite their efficacy, neuroimaging methods have several methodological disadvantages. Differences in imaging methods, sample size, and appropriate statistical approaches in organisation of the research results complicate amalgamation and analysis [31]. Another thing that standardised imaging processes and analytic methodologies would support is the comparability of research, hence making meta-analyses more reliable [43]. All necessary measures must be taken to eliminate the problem of publication bias and to develop a complete understanding of neurobiological markers. Such initiatives cannot fail to encourage research that makes the field more accurate and representative [2].

One must also pay attention to the ethical issues associated with this neural research. The ethical issues emerge from the risks of stigmatisation and the implications of employing neuromolecular knowledge in juridical plight. Prospective researchers should attempt to anticipate ethical issues by ensuring that participant anonymity and privacy are protected while sharing findings from their research in a way that is sensitive and balanced. Collaborative efforts among neuroscientists, ethicists, and legal scholars can help create ethical ideals and structure [3].

Some potential future research topics requiring mature neuroscience insights include studying the dynamic neuromolecular changes in criminal behaviour, personality disorders, and mental health illness [44]. The reason why this would support our understanding is because longitudinal studies, where people are followed during their susceptibility to a condition, can often enable us to understand the temporal changes in neurobiological markers [4]. Through the combination of multimodal strategies, including genetic analysis alongside modern neuroimaging techniques, a further understanding of the interaction between genetics and a neurological unit can be obtained [45].

In addition, various previous research-based investigations have been conducted on populations that are alike, and in most cases, the researchers eliminate certain age groups, ethnicities, or clinical subsets. Future researchers should focus on enhancing the broad applicability of findings, with relevance to understanding a larger population and improving the external validity of neural markers [3].

With regards to the development of technology, advanced neuroimaging technologies have been more accurate in imaging and brain-mapping techniques, whereas genetic research skills are improving dramatically [4]. All these developments resulting from technological improvements provide favourable prospects for future research. Using more sophisticated techniques such as functional connectivity analysis and epigenetic studies, new and important insights can be gained with respect to the neurobiology of criminal behaviour, personality disorders, and mental health, which cannot be achieved using traditional approaches [5].

Finally, the author concludes that to correctly interpret new discoveries in neurobiology, it is necessary to consider some methodological limitations that are revealed after analysis of all the existing research concerning criminal behaviour, personality disorders, and mental health issues [46]. It is recommended that future researchers strive to reach methodological relevance through the utilisation of large and representative samples as well as the widespread use of standardised techniques and ethical issues [47]. By employing cross-cutting approaches and interdisciplinary practices, we can improve our understanding of these complicated events and their dynamics [8]. To address the methodological challenges that have hindered progress in understanding neurobiological markers and to create a foundation for more efficient interventions and personalised treatments in the areas of criminal behaviour, personality disorders, and mental health, there is a need to overcome these methodological stumbling blocks and adopt advanced technologies.

## Conclusions

Anatomical alterations are observed in the PFC, and limbic system disturbances such as those of the amygdala have been characteristic of neuromolecular investigations of criminal behaviour. Hence, in this study, we propose an intricate interaction among deficits in impulse control, difficulties in emotion regulation, and abnormalities in incentive processing. Therefore, neural markers are of great importance in terms of personality disorders because they provide information related to the diversification within personality disorders and guide the development of special treatment strategies. The identified neurological basis of attitudes towards control, cognition dysfunction, and emotional dysregulation justifies developing therapies aimed at addressing these conditions. In addition, acknowledgement of genetic elements highlights the possibility of accuracy-based medicine modalities facilitating individualised treatment approaches based on an individual’s genetic material.

Furthermore, our narrative appraisal enhances the methodology by highlighting regular limitations and indicating potential solutions for further research. Various differences in sample features, imaging techniques, and diagnostic criteria are reflective of the key need for standardised methods in neural research. The theoretical approach provides methodological guidelines on how discipline can be improved by pointing out that large-scale collaborative initiatives, longitudinal designs, and multimodal evaluations are essential for this purpose. The ethical difficulties identified, including the risk of stigmatisation and use of the neurobiological data in legal settings, signify the commitment to responsible research practices.
